# Sero-prevalence of measles and rubella immunoglobulin G serum antibody in individuals 1–30 years old in England in 2018: implications for subsequent outbreaks prediction

**DOI:** 10.1007/s15010-025-02630-9

**Published:** 2025-08-28

**Authors:** Khitam Muhsen, Yoon Hong Choi, Jemma Walker, Nick Andrews, Helen I. McDonald, Kevin Brown, Elizabeth Miller

**Affiliations:** 1 Department of Epidemiology and Preventive Medicine, School of Public Health, Gray Faculty of Medical and Health Sciences, Tel Aviv University, Ramat Aviv, Tel Aviv, 6139001 Israel; 2 London School of Hygiene and Tropical Medicine, London, UK; 3 Statistics, Modelling and Economics Department, Modelling Division, UK Health Security Agency, London, UK; 4 Immunisation & Vaccine-Preventable Diseases, UK Health Security Agency, London, UK; 5 Department of Life Sciences, University of Bath, Bath, UK

**Keywords:** Measles, rubella, Seroepidemiology, England, vaccination, Effective reproduction number

## Abstract

**Purpose:**

Measles outbreaks have occurred across England since mid-2023. We estimated measles and rubella antibody seroprevalence among individuals in 2018 in English regions outside London, and estimated the effective reproduction number (Re) for measles to predict the potential for outbreaks.

**Methods:**

Using validated enzyme-linked immunosorbent assays, anti-Measles and anti-Rubella IgG antibodies were measured in residual sera from 3758 1-30-year-olds born after introduction of measles-mumps-rubella vaccination who submitted samples to clinical laboratories outside London. The measles Re was calculated using seronegatives defined by the manufacturer’s cutoff, mixture modelling, and vaccination coverage data.

**Results:**

Using the manufacturer’s cutoffs, the overall proportion seronegative to measles was 9.2% (95% confidence interval 8.3–10.1), and 10.3% (9.4–11.3) had equivocal results. The respective estimates for rubella were lower at 5.2% (4.6-6.0) and 5.4% (4.7–6.1). For both viruses, equivocal proportions increased with age, consistent with antibody waning. Mixture modelling for measles identified a common seronegative distribution across age groups, with lower proportions seronegative than using the manufacturer’s cutoff. Re for measles using the manufacturer’s seronegative cutoff (~ 150 mille international units/mL) was 1.00, versus 0.38 and 0.51 using the mixture model and vaccination coverage, respectively.

**Conclusions:**

Re for measles estimated from seroepidemiology using an antibody cut-off similar to that considered a correlate of protection for measles was a more accurate predictor of recent measles resurgences outside London than those estimated using mixture modelling of seronegatives or coverage data. Seroepidemiological studies are a useful adjunct to coverage data in monitoring population immunity and in predicting the potential for measles outbreaks.

**Supplementary Information:**

The online version contains supplementary material available at 10.1007/s15010-025-02630-9.

## Introduction

Measles is a highly infectious viral disease that can cause severe illness and even death [1, 2]. Rubella is usually a mild viral childhood disease, but infection during pregnancy can cause severe fetal malformations known as congenital rubella syndrome [3, 4]. The introduction of measles and rubella vaccines has substantially reduced the burden of these illnesses worldwide with the ultimate goal of the World Health Organisation [WHO] being the elimination of both diseases [5]. Despite an 84% reduction in globally reported measles cases between 1990 and 2019, no WHO region has achieved and sustained measles elimination [6, 7]. For rubella, which is less transmissible than measles [8], elimination was verified in 98 (51%) of 194 countries by 2022 [9].

In the United Kingdom (UK), a single-dose measles immunization program was introduced in 1968, with a selective rubella vaccination program for schoolgirls and women of childbearing age introduced in 1970. The single antigen measles vaccine was replaced by a combined measles, mumps, and rubella (MMR) vaccine in 1988 [2] to be given at around one year of age. In 1994, a nationwide measles/rubella (MR) catch-up immunization campaign for school-aged children was conducted, followed in 1996 by the introduction of a second MMR dose before school entry. High uptake for the MMR programme and the 1994 MR campaign successfully interrupted measles and rubella transmission [2, 3]. However, MMR coverage declined in the late 1990s and early 2000s following false claims that the MMR vaccine caused autism, and low-level measles transmission was re-established in 2006 [2]. MMR catch-up campaigns were conducted in London in 2004/5 for children up to five years of age and nationwide in 2008 for those up to 18 years and again in 2012/13 for teenagers. By 2014, the UK had again interrupted measles transmission, but subsequent declines in vaccination coverage contributed to the re-establishment of measles transmission in 2018 [2]. Rubella elimination in the UK was confirmed in 2015 and has been sustained [10].

To assess the potential for sustained transmission and the need for catch-up MMR campaigns, the UK Health Security Agency (UKHSA) uses vaccine coverage data to estimate age-specific susceptibility and the effective reproduction number (Re) for different geographical areas in England [11]. Re represents the number of secondary infections that will occur in a population after the introduction of a single infectious case; a value of 1 or greater indicates the potential for large outbreaks and sustained transmission. When estimated in 2023, as measles cases increased after lifting of the COVID-19 restrictions on social mixing, the risk of a large measles epidemic outside of London was estimated to be low (Re < 1), though not in London (Re > 1), where coverage had been consistently below the national average [12]. However, there was a large rise in cases in 2024 in England, with more than half of the confirmed cases in 2024 and the first half of 2025 occurring outside London [13].

The use of historical coverage data to estimate age-specific immunity is usually considered to result in an underestimate of immunity due to incomplete recording on child health information systems of vaccinations given in primary care [14, 15]. Seroepidemiology provides an alternative way of estimating population immunity, assuming the relationship between antibody level and protection is known. The aim of this study was to examine the prevalence of serum IgG antibodies against rubella and measles in regions outside London using sera collected in 2018 from individuals born after MMR introduction in whom immunity is predominantly vaccine-induced. We estimate measles and rubella antibody prevalence utilizing both the recommended cut-off values for the antibody assay and a mixture modelling approach, which can identify different antibody distributions in the population. We compare Re estimates for measles based on serology with those derived from coverage data [16].

## Materials and methods

### Study design and population

Residual sera from samples taken in 2018 from 3761 individuals aged 1–30 years submitted to laboratories in England for microbiological or chemical pathology were analysed. As part of national disease prevention programs, the UKHSA runs periodical serosurveys to assess the population’s immunity to vaccine-preventable diseases utilizing residual sera [16–19].

Residual sera were collected by the UKHSA Seroepidemiology Unit from six laboratories in England that served populations in the following English regions: Yorkshire and Humberside, South West, North East, North West, and East Midlands.

A sample size of 522 individuals gives a 95% confidence interval (CI) up to 6% wide (i.e., 3% precision), assuming at least 90% seropositivity. The available sample size of 3761 was expected to enable such assessment in six age groups (1–4, 5–9, 10–14, 15–19, 20–24, 25–30).

The dataset included information on age in years, gender, and region (independent variables), as well as measles and rubella serology results.

#### Outcome measures

Prevalence of serum IgG antibodies against measles and rubella. 

Both anti-measles and anti-rubella IgG antibodies were measured using the validated Siemens Enzygnost^®^ anti-Measles virus and anti-Rubella-Virus enzyme-linked immunosorbent assay (ELISA) kits [20, 21], respectively, following the manufacturers’ instructions and recommended cutoff points to determine serostatus. Specimens with optical density (OD) values less than 0.1, between 0.1 and 0.2, and above 0.2 were considered seronegative, equivocal, and seropositive, respectively. Alternative cutoff values were generated using the mixture modelling approach (details in the data analysis section). A validation study of the kit in the framework of the European Sero-Epidemiology Network project demonstrated high sensitivity and specificity and agreement in repeated measurements (R^2^ > 0.8). The sensitivity and specificity of the ELISA kit versus the gold standard, plaque neutralization test (a functional antibody assay that measures the neutralization activity of antibodies), have previously been estimated at 91.6%-95.3% and 96.0%-100.0%, respectively, for measles [20], and 100.0% and 92.8%, respectively, for rubella [21]. The test kit OD cutoff of 0.1 corresponded to a measles antibody concentration of around 150 mille international units/mL (mIU/mL) as estimated using the reference serum in the Enzynost assay, with the OD cutoff for seropositivity being around 300 mIU/mL. The correlate of protection for measles, measured by the gold standard plaque reduction neutralisation test, is generally accepted to be around 120 mIU/mL [22, 23].

Information on measles and rubella notifications and vaccination coverage was obtained from the publicly available data on the NHS England website [24].

### Data analysis

#### Using the manufacturer’s recommended OD cutoff values

We calculated the overall seroprevalence proportion (and 95% CI) and the proportion by age group, sex, and region of individuals with positive, equivocal, and negative test results for measles and rubella IgG antibodies. Differences in measles and rubella serostatus according to demographic factors were examined using the chi-square test. Logarithm (Log10) transformation was performed for the OD values of the measles and rubella serology results as a proxy for the magnitude of the immune response among seropositive individuals. The student’s *t*-test and one-way analysis of variance (ANOVA) were used to assess differences in the mean log10 OD of measles and rubella IgG values of seropositive individuals by sex, age, and region, and multiple linear regression models assessed adjusted associations. Results were anti-logged to give geometric means and fold differences between groups.

#### Mixture modelling

Mixture modeling was applied to the log10 transformation of the OD values of the measles and rubella serology data to assess the suitability of the manufacturer’s recommended cut-off for negativity [16].

Histograms were used to inspect the distribution of log10 of measles and rubella IgG ODs by age group (1–4, 5–9, 10–14, 15–19, 20–24, and 25–30 years) and suggested that a good fit would be obtained using a common negative normal distribution across all ages and a mixture of low positive and high positive normal distributions which varied with age. We explored a two-component mixture model (one positive and one negative) and employed the Bayesian Information Criterion (BIC) to compare the fit of the three vs. two distribution models. Using Stata (STATA Corp, TX), we estimated (on a log scale) the mean and standard deviation of the common negative distribution, the mean and standard deviation of the positive distributions (as a proxy of the magnitude of the immune response) for each age group, and the proportions in the negative, low positive, and high positive distributions in each age group. Fitting in Stata to find the parameters that maximized the log-likelihood was done using the ml functions (Supplementary material). OD values < 0.001 were censored at 0.001. Because these censored results made up a fair proportion of the negative distribution, the likelihood function was written to include these results as censored at < 0.001 rather than at a value of 0.001. This means that the fitted negative distribution, when plotted, appears as a spike at 0.001 and then a partial normal distribution on the right-hand side. We provided estimates of seronegativity based on the estimated proportion in the negative distribution. The fitted models were also used to derive assay cut-offs that gave negative proportions that best matched the modelled proportions. This was done first by having a single cut-off irrespective of age and then by allowing different cut-offs by age. This was done to enable individual results to have a status (negative or positive) assigned to them.

#### Correlations

The correlation between measles and rubella results using the OD cutoff defined by the manufacturer and as redefined by mixture modelling (using the common cut-off for all ages) was examined using Pearson’s correlation coefficient for continuous data and Cohen’s Kappa coefficient for categorical data.

#### Calculation of the effective reproduction number (Re)

The Re for measles was calculated from the Next Generation Matrix, taking into account the proportion susceptible in each age group (0–4, 5–10,11–17,18–24, ≥ 25 years) and assuming a basic reproduction number of 10.7 as previously described [11]. Age-specific susceptibility using serology data was estimated utilising the manufacturer’s OD recommended cutoff values and the results of the mixture modelling. It was assumed that infants are protected by maternal antibodies for the first six months of life and then are susceptible until receiving the MMR vaccination. For those aged 31 to 90 years, it was assumed that only 2% are susceptible, as these individuals belonged to the pre-MMR vaccination birth cohort when measles incidence was still high and almost all adults acquired immunity through infection or infection and measles vaccination in those aged 31–55 years [25]. Since the force of infection during the pre- and post-vaccination periods was shown to be very low in adults [25], Re estimates are not sensitive to the precise level of susceptibility assumed for the 31-90-year-olds.

Coverage data were also used to estimate Re in 2018 for English regions, excluding London, as described [12] with varying assumptions about the proportion of those without a recorded vaccination history that had been vaccinated (10%, 25%, and 50%). The efficacy of a dose of MMR vaccine against measles was assumed to be 95%, and 99.75% for two doses, as in previous analysis [26]. In a sensitivity analysis, an efficacy of 96% for a second dose of MMR vaccine was assumed based on the results of a recent Cochrane review [27].

Data were analysed using IBM SPSS (IBM, Armonk, New York, NY, USA) software version 28, STATA, and Microsoft Excel was used for Re estimation.

### Ethics approval

The study was conducted as a joint project between UKHSA and the London School of Hygiene and Tropical Medicine. The joint UCL/UCLH Committee on the Ethics of Human Research provided a generic approval for the use of residual anonymised serum samples, reference number 05/Q0505/45. The current analysis was approved by the Ethics Committee of the London School of Hygiene and Tropical Medicine: reference number 28,348.

## Results

Cyclic measles epidemics with thousands of cases notified occurred in England and Wales in the pre-measles vaccination years. Following the introduction of measles vaccination, the number of notified cases declined progressively as coverage increased (Fig. 1a).

**Fig. 1a:**
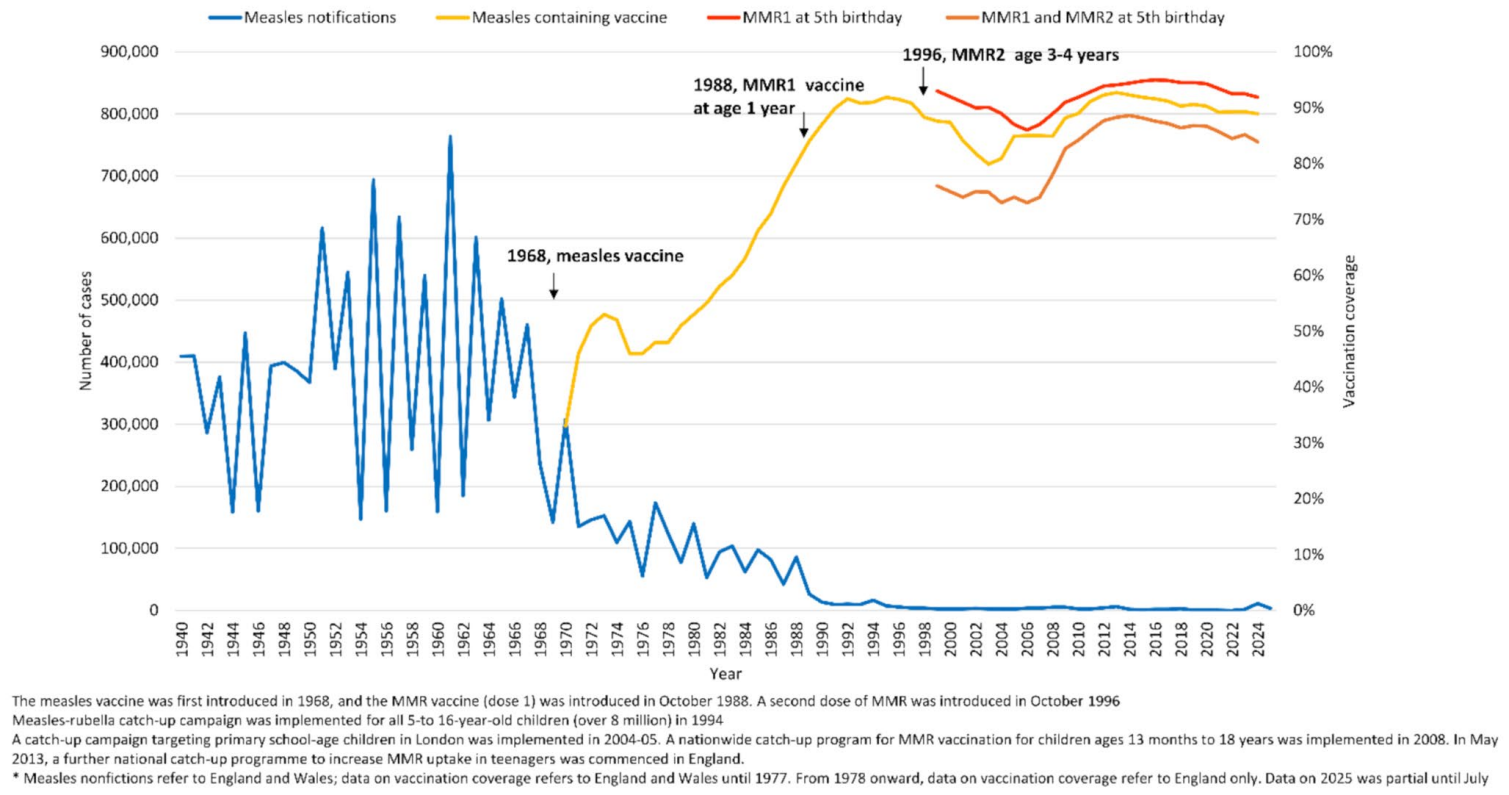
The number of measles notifications and vaccine coverage in England and Wales, 1940–2025^*^

The number of measles notifications in England and Wales increased from 53 in 2022 to 1619 in 2023, rising to 11,162 notifications in 2024, and 3268 notifications between January and July 2025 in England and Wales [28]. The number of notifications in 2024 was the highest since 1994. Of the laboratory-confirmed cases in England, the majority in early 2023 were in the London region. However, in October 2023, there was a rapid rise in measles cases due to a large outbreak in Birmingham in the West Midlands region; overall of the 367 confirmed cases in 2023, 43.3% were reported in the West Midlands, with 14.4% reported from other regions outside London [29]. In 2024, 55% of the 2911 confirmed cases were in regions outside London; of the 64 local authorities with at least 10 confirmed cases, 35 were outside London [12]. Similarly between January and July 2025 more than half of the confirmed cases were in regions outside London [30]. All age groups were affected, but the majority of cases were children aged 0–10 years (Supplementary Table 1, Supplementary Fig. 1).

Routine coverage of MMR vaccine in England has been steadily declining since 2013 (Fig. 1). London is characterised by a relatively lower MMR vaccination coverage (86.1% for first dose by the age of 5 years in 2022–2023) than other regions in England (92.6%-95.5%) [24].

Fig. 1b shows the consistent decline in the number of rubella notifications, with only travel-associated or important cases detected in recent years.

**Fig. 1b:**
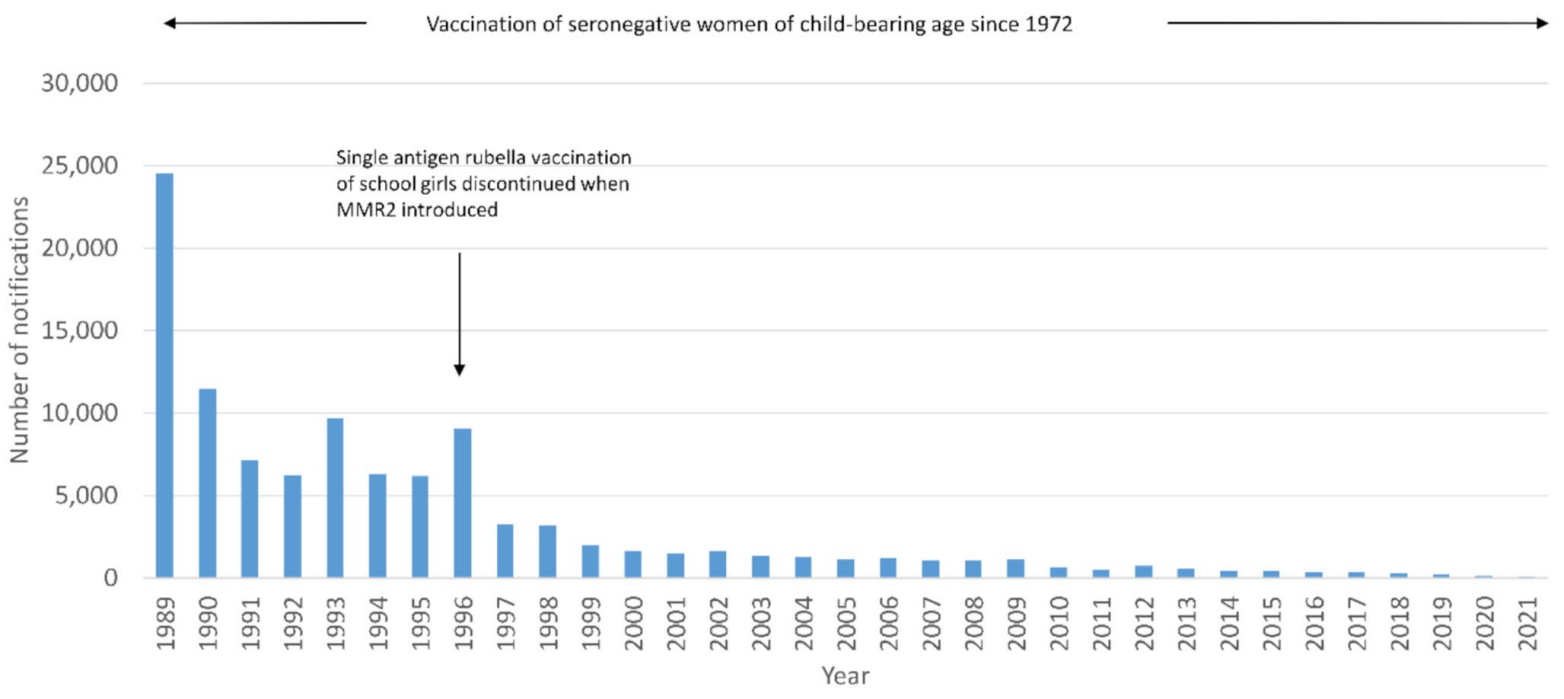
The number of rubella notifications by age group in England and Wales, 1989–2021

### Seroepidemiological study

Out of 3761 available sera, three were excluded from the analysis due to missing data (*n* = 1 lacked results of rubella and measles serology results, *n* = 1 out of the age range, and *n* = 1 lacked information on gender), thus leaving in the analysis sera from 3758 individuals (56.2% females) aged 1–30 years (mean of 17.1 years (SD = 8.1)). Most (51.7%) participants were from the North West region, followed by Yorkshire and Humberside (17.9%), South West (15.1%), North East (13.0%), and only 2.4% were from East Midlands (Supplementary Table 2).

### Measles IgG antibody seroprevalence

Using the manufacturer’s recommended OD cutoff value, there were 3027 (80.5%) individuals with a positive result for measles IgG antibody, with the highest proportions positive in 1-4- and 5–9-year-olds, 90.5% and 89.9%, respectively (Table 1). The percentage of individuals with equivocal results increased with age until 25–30 years. Among seropositive individuals, the geometric mean titer of measles IgG level was highest in 1-4-year-olds and decreased with age (Supplementary Table 3). Females had a higher seropositivity than males, 82.0% vs. 78.7% (*P* = 0.012). No significant regional differences were found in measles serostatus (*P* = 0.092).

**Table 1:** Sero-prevalence of measles IgG antibody according to demographics, England 2018, based on the manufacturer’s recommended OD cut-off value ^#^

Variable	Total	Negative		Equivocal		Positive		*P* value*
		*N*	% (95% CI)	*N*	% (95% CI)	*N*	% (95% CI)	
**Overall**	3758	344	9.2% (8.3–10.1)	387	10.3% (9.4–11.3)	3027	80.5% (79.3–81.7)	
**Age**,** years**								< 0.001
1–4	358	31	8.7% (6.2–12.0)	3	0.8% (0.3–2.4)	324	90.5% (87.0-93.1)	
5–9	514	23	4.5% (3.0-6.6)	29	5.6% (4.0–8.0)	462	89.9% (87.0-92.2)	
10–14	587	66	11.2% (8.9–14.1)	62	10.6% (8.3–13.3)	459	78.2% (74.7–81.4)	
15–19	773	75	9.7% (7.8–12.0)	95	12.3% (10.2–14.8)	603	78.0% (75.0-80.8)	
20–24	769	75	9.7% (7.9–12.1)	107	13.9% (11.7–16.5)	575	74.8% (71.6–77.7)	
25–30	757	62	8.2% (6.4–10.4)	91	12.0% (9.9–14.5)	604	79.8% (76.8–82.5)	
**Sex**								0.012
Males	1645	175	10.6% (9.2–12.2)	176	10.7% (9.3–12.3)	1294	78.7% (76.6–80.6)	
Females	2113	169	8.0% (6.9–9.2)	211	10.0% (8.8–11.3)	1733	82.0% (80.3–83.6)	
**Region**								
East Midlands	90	7	7.8% (3.8–15.2)	15	16.7% (10.4–25.7)	68	75.6% (65.8–92.3)	0.092
North East	487	45	9.2% (7.0-12.1)	48	9.9% (7.5–12.8)	394	80.9% (77.2–84.2)	
North West	1943	193	9.9% (8.7–11.3)	197	10.1% (8.9–11.6)	1553	79.9% (78.1–81.7)	
South West	566	57	10.1% (7.9–12.8)	61	10.8% (8.5–13.6)	448	79.2% (75.6–82.3)	
Yorkshire and Humberside	672	42	6.3% (4.7–8.3)	66	9.8% (7.8–12.3)	564	83.9% (81.0-86.5)	

CI: confidence interval; IgG: immunoglobulin G; OD: optical density

^#^ Negative: Optical density (OD) < 0.1, equivocal: OD from 0.1 to 0.2, and positive: OD > 0.2 using the manufacturer’s recommended cut-off values

* *P* value was obtained by the chi-square test

### Mixture modelling of measles IgG antibody seroprevalence

The distributions of the log10 of the measles IgG ODs are shown by age in Supplementary Fig. 2, suggesting the existence of three distributions as outlined above. The BIC results of the three-distribution and two-distribution models were 4835.2 and 4918.3, respectively, consistent with a better model fit for the three-distribution model.

As age increases, the distributions shift to the left, with an increasing proportion of individuals with low OD values above the 0.001 minimum OD but below the manufacturer’s recommended OD cutoff for negativity of 0.1. This suggested the waning of antibody levels as time since vaccination increased, with a common negative OD distribution across age groups and low and high positive OD distributions that varied with age. The mean log10 OD of measles IgG antibody in both positive distributions decreased with age (Supplementary Table 4). The distribution of negative, low, and high positive measles IgG ODs for all ages combined is shown in Fig. 2a.

**Fig. 2a:**
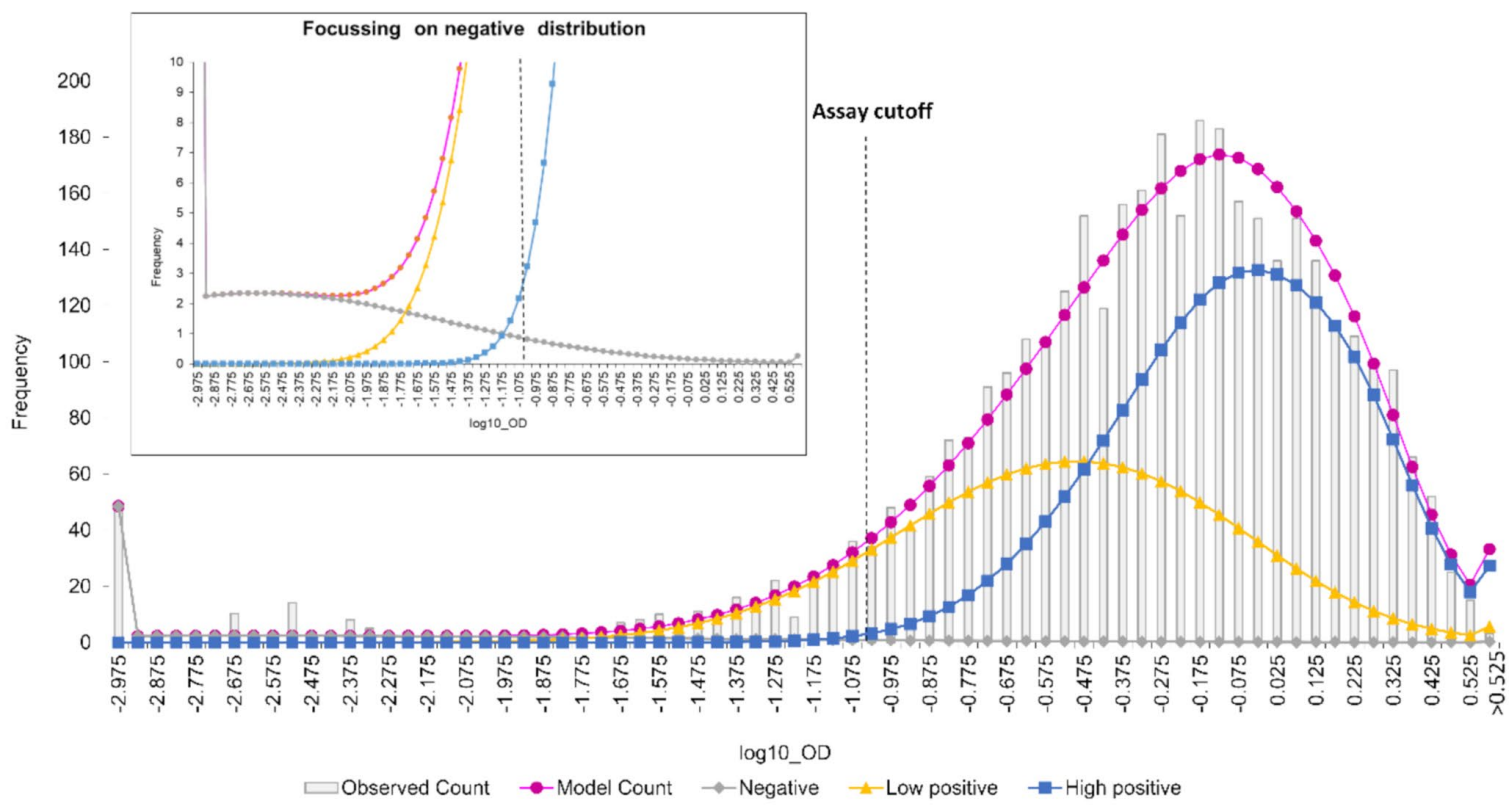
Overall measles mixture model combining all age groups

The OD cutoff that gave proportions negative closest to the mixture-modelled proportions across all ages was 0.03. When optimised within individual age groups, the OD cutoff ranged from 0.15 to 0.02 (Supplementary Table 5). The proportions seronegative in those aged five years and above were higher using the manufacturer’s OD cutoff of 0.1 than estimated using mixture modelling.

### Rubella IgG serum antibody seroprevalence

Using the manufacturer’s recommended OD cutoff value, there were 3361 (89.4%) individuals with positive test results for rubella IgG serum antibody; the highest proportions positive were in children aged 5–9 and 1–4 years, 94.8% and 91.9%, respectively (Table 2). Rubella IgG antibody seropositivity declined in the age groups 10–24 (86.1%-87.7%), but it increased to 91.8% in 25-30-year-olds. Females had a higher rubella IgG seropositivity than males: 91.2% vs. 87.1%, in addition to geographic variation (Table 2). The percentage of individuals with equivocal results increased with age until 25–30 years. Among seropositive individuals, the geometric mean titer of the IgG level decreased with age, and individuals from the North West and South West regions had slightly lower levels than those in Yorkshire and Humberside (Supplementary Table 6).

**Table 2:** Sero-prevalence of rubella IgG antibody according to demographics, England 2018 based on the manufacturer’s recommended OD cutoff value ^#^

Variable	Total	Negative		Equivocal		Positive		*P* value*
		*N*	% (95% CI)	*N*	% (95% CI)	*N*	% (95% CI)	
**Overall**	3758	196	5.2% (4.6-6.0)	201	5.4% (4.7–6.1)	3361	89.4% (88.4–90.4)	
**Age**,** years**								< 0.001
1–4	358	25	7.0% (4.8–10.1)	4	1.1% (0.4–2.8)	329	91.9% (88.6–94.3)	
5–9	514	16	3.1% (1.9-5.0)	11	2.1% (1.2–3.8)	487	94.8% (92.5–96.4)	
10–14	587	31	5.3% (3.8–7.4)	41	7.0% (5.2–9.3)	515	87.7% (84.8–90.2)	
15–19	773	47	6.1% (4.6-8.0)	53	6.9% (5.3–8.9)	673	87.0% (84.5–89.3)	
20–24	769	46	6.0% (4.5–7.9)	61	7.9% (6.2–10.1)	662	86.1% (83.5–88.4)	
25–30	757	31	4.1% (2.9–5.8)	31	4.1% (2.9–5.8)	695	91.8% (89.4–93.6)	
**Sex**								< 0.001
Males	1645	112	6.8% (5.7–8.1)	100	6.1% (5.0-7.3)	1433	87.1% (85.4–88.7)	
Females	2113	84	4.0% (3.2–4.9)	101	4.8 (4.0-5.8)	1928	91.2% (90.0-92.4)	
**Region**								
East Midlands	90	6	6.7% (3.1–13.8)	4	4.4% (1.7–10.9)	80	88.9% (80.7–93.9)	0.005
North East	487	23	4.7% (3.2-7.0)	19	3.9% (2.5-6.0)	445	91.4% (88.6–93.6)	
North West	1943	111	5.7% (4.8–6.8)	119	6.1% (5.1–7.3)	1713	88.2% (86.7–89.5)	
South West	566	37	6.5% (4.8–8.9)	35	6.2% (4.5–8.5)	494	87.3% (84.3–89.8)	
Yorkshire and Humberside	672	19	2.8% (1.8–4.4)	24	3.6% (2.4–5.3)	629	93.6% (91.5–95.2)	

CI: confidence interval; IgG: immunoglobulin G

^#^ Negative: Optical density (OD) < 0.1, equivocal: OD from 0.1 to 0.2, and positive: OD > 0.2 using the manufacturer’s recommended cutoff values

* *P* value was obtained by the chi-square test

### Mixture modelling of rubella IgG antibody seroprevalence

Inspection of the rubella OD distributions similarly suggested a common negative distribution, and low and high positive distributions that varied with age (Supplementary Fig. 3). The distributions of the negative, low, and high positive rubella IgG ODs are shown in Fig. 2b for all age groups combined. The OD cutoff that gave proportions negative closest to the mixture-modelled proportions across all ages was 0.02. When optimised within individual age groups, the OD cutoff ranged from 0.1 to 0.01 (Supplementary Table 7). The proportions that were seronegative in those aged five years and above were substantially higher using the manufacturer’s OD cutoff of 0.1 than estimated using mixture modelling.

**Fig. 2b:**
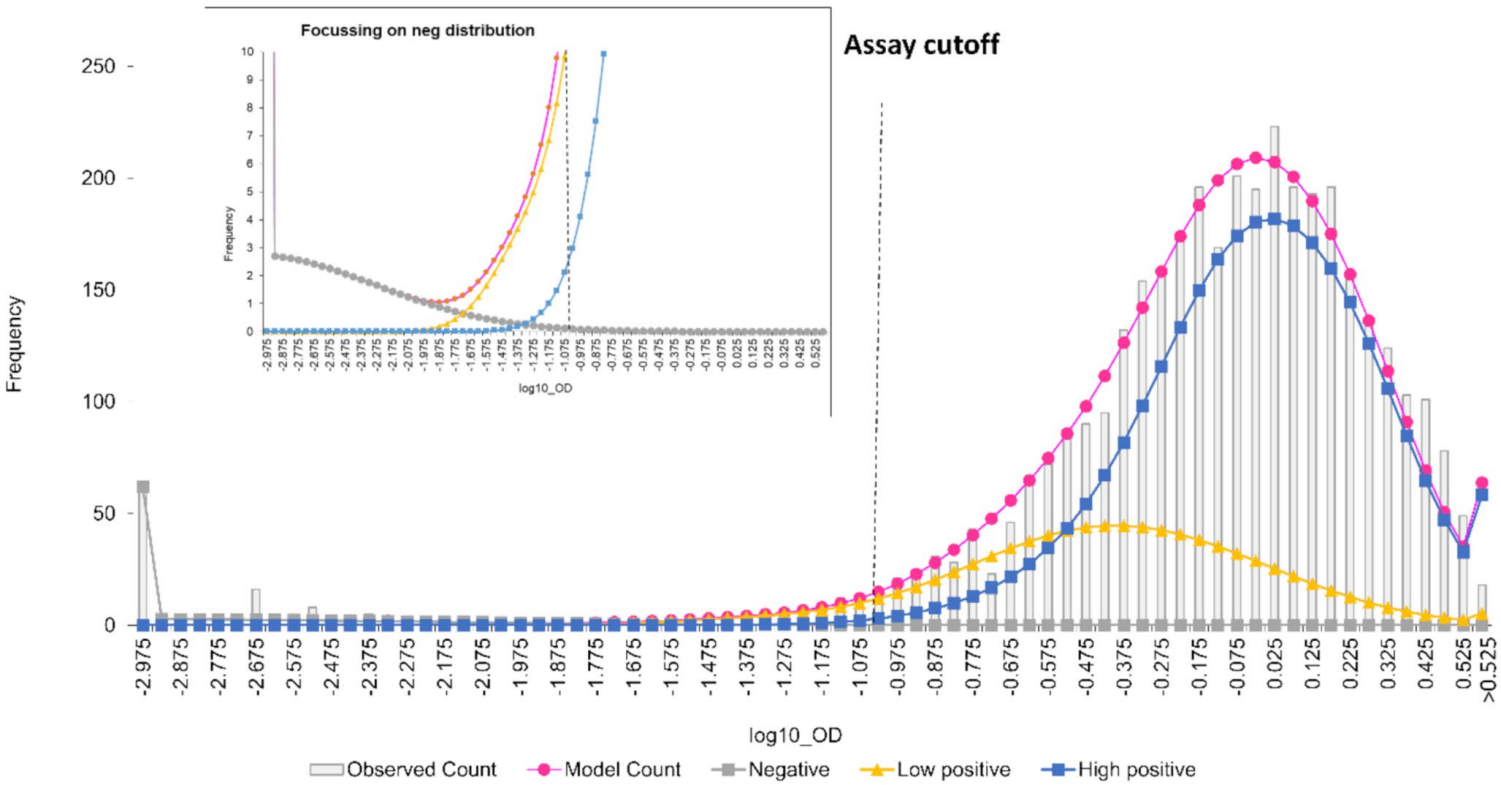
Overall rubella mixture model combining all age groups

### Correlation between rubella and measles IgG seroprevalence

There was a significant correlation between measles and rubella IgG antibody levels: Pearson’s correlation coefficient 0.656, *P* < 0.001 (Supplementary Fig. 4). Using the manufacturer’s OD < 0.1 cutoff to determine seronegativity and combining the positive and equivocal results yielded 92.5% overall concordance between rubella and measles IgG antibody serostatus, with 89.1% and 3.4% of the samples being seropositive and seronegative for both antigens, respectively (Table 3). Cohen’s Kappa coefficient was 0.44 (95% CI 0.41–0.47). The overall concordance was 97.9% when using the mixture models’ cutoff OD < 0.03 for measles and OD < 0.02 for rubella, with 95.7% and 2.2% of the samples being classified as positive and negative for both antigens, respectively. Cohen’s Kappa coefficient was 0.66 (95% CI 0.63–0.70).

**Table 3:** Concordance between measles and rubella IgG seroprevalence, England 2018

A: Using the manufacturer’s OD < 0.1 cut-off to determine seronegativity (positives include equivocal results)						
		Rubella IgG seroprevalence			Overall agreement	Cohen’s Kappa coefficient (95% CI)
		Negative	Positive	Total		
Measles IgG seroprevalence	Negative	**129 (3.4%)**	215 (5.7%)	344 (9.2%)	**92.5%**	**0.44** **(0.39–0.49)**
	Positive	67 (1.8%)	**3347 (89.1%)**	3414 (90.8%)		
	Total	196 (5.2%)	3562 (94.8%)	3758 (100.0%)		

B: Using mixture models best cut-off OD < 0.03 for measles and OD < 0.02 for rubella						
		Rubella IgG seroprevalence			Overall agreement	Cohen’s Kappa coefficient (95% CI)
		Negative	Positive	Total		
Measles IgG seroprevalence	Negative	**83 (2.2%)**	54 (1.4%)	137 (3.6%)	**97.9%**	**0.66 (0.59–0.73)**
	Positive	26 (0.7%)	**3595 (95.7%)**	3621 (86.4%)		
	Total	109 (2.9%)	3649 (97.1%)	3758 (100.0%)		

CI: confidence intervals; IgG: immunoglobulin G; OD: optical density

### Estimating the effective reproduction number (Re)

The Re value in 2018, as estimated from serology data using the manufacturer’s OD recommended cutoff value for seronegativity and assuming that those in the equivocal range were immune, was 1.00 (Table 4). Assuming that those with equivocal results were susceptible increased the Re value to 2.24, whereas using the mixture model negative distribution, the Re was 0.38 (Table 4). The Re value, as estimated from historical coverage data up to 2018, was 0.51 (Table 4). This assumed that 50% of those without a documented vaccine history were vaccinated. Assuming that 10% or 25% of those without a documented history of MMR are vaccinated, then the Re value is higher at 0.65 and 0.57, respectively (Supplementary Table 8). The Re assuming 96% efficacy estimate for the second dose falls within the range 1.12 to 0.80, depending on whether it is assumed that 10% or 50%, respectively, of those without a documented MMR history have been vaccinated (Supplementary Table 8).

**Table 4:** Estimates of the effective reproductive number in 2023 using the serology results under different scenarios to determine the percentage of susceptible individuals in each age group and from COVER data for England excluding London

Age group, years	Negative serology results only (equivocal are considered positive) ^a^	Negative and equivocal serology results are combined ^a^	Mixture model negative distribution	Estimated from coverage data ^b^
0–4	18.0%	18.8%	17.5%	16.9%
5–10	4.4%	9.7%	2.2%	3.0%
11–17	10.8%	21.9%	5.8%	3.9%
18–24	10.9%	25.0%	2.3%	5.3%
25–90	2.6%	3.7%	0.9%	2.7%
**Re**	**1.00**	**2.24**	**0.38**	**0.51**

^a^ Using the manufacturer’s cut-off values to determine susceptibility

^b^ Using COVER data up to 2018 and assuming 50% of those with 0 record of MMR are vaccinated

## Discussion

We examined measles and rubella IgG antibody seroprevalence in a large sample of residual sera collected in 2018 from individuals aged 1–30 years who were born after the introduction of MMR vaccination in England. This cohort had little exposure to measles and rubella as transmission in England had been substantially reduced since the early 1990s by high MMR vaccine coverage and the pre-school MMR catch-up programme conducted in 1988 when MMR was introduced [2]. The antibodies detected in the study cohort were, therefore, predominantly derived from vaccination rather than infection, except for some individuals in the 25-30-year age group.

Using the manufacturer’s OD cutoff values, the overall proportion seronegative to measles was 9.2%, with a further 10.3% having low antibody levels categorised as equivocal. The Re was substantially different depending on whether those with antibody levels in the equivocal range were considered susceptible or immune to measles, 2.24 and 1.00, respectively. The test kit OD cutoff of 0.1 corresponded to a measles antibody concentration of around 150 mille international units/mL (mIU/mL) as estimated using the reference serum in the Enzynost assay, with the OD cutoff for seropositivity being around 300 mIU/mL. The correlate of protection for measles measured by the gold standard plaque reduction neutralisation test is generally accepted to be around 120 mIU/mL [22, 23], suggesting that the Re value based on assuming those with antibodies in the equivocal range are protected is the more accurate. However, there are well-documented instances of individuals with pre-exposure neutralisation titres substantially greater than 120 mIU/mL who have developed clinical measles. Indeed, it has been proposed that the relationship between measles antibody level and protection is better described as one in which the risk of infection is reduced with increasing antibody levels rather than there being a threshold level above which protection is absolute [22].

The mixture modelling approach identified an OD cutoff for seronegativity of 0.03, which was substantially lower than that recommended by the test kit OD cutoff (0.1). However, the Re estimate using only individuals with OD values in the modelled negative distribution (0.38) would seem inconsistent with the observed level of measles transmission. This would suggest that some individuals in the low positive distribution were not protected against measles infection. However, there was no evidence of a separate distribution within the low positives that might constitute a susceptible group and regarding all low positives as susceptible is unrealistic given the high proportion of individuals in this distribution. The mixture modelling approach was therefore uninformative in relating antibody levels to protection.

The Re value in 2018 for regions outside London, estimated using coverage data, was substantially lower than that calculated from the serological data with the manufacturer’s cut-off, ranging from 0.51 to 0.65, depending on the assumptions made about under-ascertainment of coverage. When recalculated in 2023 using updated coverage estimates, similar low Re values were obtained [12], suggesting that susceptibility to measles outside London is well below the threshold for large measles outbreaks or sustained transmission to occur. In contrast, the Re value of 1.00 from the serological data using the manufacturer’s cutoff for negativity indicated a susceptibility level around the endemic threshold and a risk of large outbreaks. The increasing incidence of measles cases in regions outside London between 2023 and mid-2025, following the very low levels between 2020 and 2022 during the COVID-19 pandemic [13], suggests that the Re estimated from serological data with the manufacturer’s cut-off may be the more realistic estimate. In theory, when Re is < 1, each measles case will produce less than one secondary case, and the number of cases will decrease. However, in real life, relatively large outbreaks with hundreds of cases can still occur when Re is < 1, due to variations in susceptibility and mixing patterns, and importation risk. To account for this heterogeneity, the WHO susceptibility targets to achieve elimination were set to maintain Re < 0.7 [31, 32]. The Re estimates using the manufacturer’s cutoff values exceeded the WHO target and indicated the potential for large measles outbreaks in regions outside London, as occurred in 2023–2025, and which posed a significant public health risk [33].

The key assumptions that affect Re values for measles estimated using coverage data are the basic reproduction number, for which the same value (10.7) was assumed for the estimates from coverage and serological data, and the assumed efficacy of one and two doses of vaccine. In the UKHSA analyses [12, 26], the efficacy of a single MMR dose was assumed to be 95% and 99.75% for the second dose, based on the assumption that vaccine failure to any dose is random, so that 95% of the remaining 5% of primary vaccine failures will seroconvert after a second dose. However, a Cochrane review in 2021 provided dose-specific efficacy estimates of 95% and 96% for first and second doses, respectively [27]. In the sensitivity analysis in which 96% efficacy for the second dose was assumed [33], the Re values ranged between 1.12 and 0.80, depending on the assumption about the proportion of vaccinated individuals (10% or 50%) among those without a documented MMR history. These Re estimates exceed the WHO-recommended Re value and may be the more realistic estimates.

In our study, the proportions of individuals with measles antibody levels in the equivocal range increased with age, consistent with vaccine-induced antibody levels waning with time since vaccination [34, 35]. This was supported by the linear regression model limited to seropositive individuals, which showed a decrease in OD values with increased age up to 20–24 years (Supplementary Table 3). The mixture modelling analysis suggested three distributions for measles antibodies: a common negative distribution across all ages and low and high positive distributions that varied with age. The negative distribution likely represents individuals who are immunologically naïve, never having been infected or vaccinated, or if vaccinated, have failed to mount any antibody response. In contrast, the mean values of the log10 OD of measles IgG antibody in the low and high positive distributions decreased with age (Supplementary Table 4), consistent with antibody waning with time since vaccination. The extent to which waning measles antibody levels after vaccination contribute to lack of protection is unclear, but indirect evidence of waning protection against measles after successful vaccination (i.e. secondary vaccine failure) was obtained by Robert et al. [36] who compared the proportion vaccinated and the age distribution of vaccinated cases among laboratory-confirmed measles cases in England between 2010 and 2019 with those expected used a compartmental transmission model. The results were consistent with a slow rate of waning protection (0.39% per year) since the cessation of endemic transmission [36]. Waning of immunity after successful measles vaccination has the potential to increase the number of cases, particularly among teenagers and young adults, when outbreaks occur in near-elimination settings, though its contribution to sustaining endemic transmission is unclear.

Using the manufacturer’s cutoff values, the overall rubella IgG seropositivity was 89% among 1-30-year-olds, which is above the level needed for interrupting rubella transmission and achieving elimination [8]. The seropositivity rate for rubella was higher than for measles, and while some of the older females in our study population may have received additional doses of rubella-containing vaccines through pre-pregnancy or post-partum vaccination, rubella seropositivity was still around 4% higher than measles seropositivity among males. This is consistent with the higher seroconversion rates for rubella than the measles component of the MMR vaccine [35]. The high concordance between serostatus for measles and rubella (Table 3) is expected, given that the antibodies in the study cohort are both largely derived from the MMR vaccine.

Our sero-epidemiological study has limitations, mainly the representativeness of our sample was limited due to using a convenience sample of residual sera from clinical laboratories. These samples were obtained from individuals with access to healthcare services whose healthcare-seeking behaviours might be related to vaccination status. The sample was also geographically limited as it did not include laboratories from all eight regions outside London, nor did it include samples from London, where Re estimates based on coverage data have suggested the potential for large outbreaks depending on the assumptions made about underascertainment of coverage [12]. In addition, within any one region, MMR coverage will not be uniform and is likely to be lower in densely populated areas and those with high levels of deprivation [37]. This heterogeneity has been reflected in the pattern of measles outbreaks in 2023 and 2024, which have tended to cluster in such communities. Seroepidemiological studies focusing on under-vaccinated communities are needed to better understand the pockets in the population’s immunity and predict potential outbreaks, alongside interventions for increasing MMR vaccination coverage [38].

## Conclusions

This large serosurvey using sera collected in 2018 from individuals born after the introduction of MMR vaccination in England suggested the potential for large outbreaks of measles in regions outside London, consistent with the resurgence of measles seen following resumption of mixing after the COVID-19 pandemic in England. Given the uncertainty inherent in coverage estimates and despite some uncertainty in relating measles antibody levels to susceptibility, seroepidemiological studies can provide useful insights into population immunity and progress toward measles elimination.

## Supplementary Information

Below is the link to the electronic supplementary material.

Supplementary Material 1

## Data Availability

The dataset cannot be made publicly available due to regulatory restrictions.
