# Supplementary material for: Sero-prevalence of measles and rubella immunoglobulin G serum antibody in individuals 1–30 years old in England in 2018: implications for subsequent outbreaks prediction
Source: Infection. 2025 Aug 28;53(6):2809–20. doi: 10.1007/s15010-025-02630-9 (PMC12675733; doi:10.1007/s15010-025-02630-9)
Supplement: Supplementary file 1 — Supplementary Material 1 [file 15010_2025_2630_MOESM1_ESM.pdf]

Sero-prevalence of measles and rubella immunoglobulin G serum antibody in individuals 1-30 years old in England in 2018: implications for subsequent outbreaks prediction

Khitam Muhsen[1,2#] ((ORCID number 0000-0003-1086-7559), Yoon Hong Choi [3], Jemma Walker [4], Nick Andrews [4], Helen I. McDonald [2,5], Kevin Brown [4], Elizabeth Miller [2]

[1] Department of Epidemiology and Preventive Medicine, School of Public Health, Gray Faculty of Medical and Health Sciences, Tel Aviv University, Tel Aviv, Israel

[2] London School of Hygiene and Tropical Medicine, London, United Kingdom

[3] Statistics, Modelling and Economics Department, Modelling Division, UK Health Security Agency, London, United Kingdom

[4] Immunisation & Vaccine-Preventable Diseases, UK Health Security Agency, United Kingdom

[5] Department of Life Sciences, University of Bath, Bath, United Kingdom

Supplementary Table 1: Laboratory confirmed-cases of measles by age group and region in England 2023-July 2025.

**Laboratory confirmed-cases of measles by age group and region in England: 1 January 2023 to 31 December 2023**										
**Age group**	**East Midlands**	**East of England**	**London**	**North East**	**North West**	**South East**	**South West**	**West Midlands**	**Yorkshire and Humber**	**Total**
less than 1 year	1	0	16	0	2	2	1	21	4	47
1 to 4 years	0	2	36	1	3	5	4	39	9	99
5 to 10 years	1	3	29	1	1	2	0	46	4	87
11 to 14 years	2	0	8	0	1	1	2	23	0	37
15 to 24 years	5	2	12	0	0	1	2	9	3	34
25 to 34 years	0	0	11	0	0	1	0	12	6	30
35 years and older	0	2	13	1	1	2	1	9	4	33
Total	9	9	125	3	8	14	10	159	30	367
**Laboratory confirmed cases of measles by age group and region in England: 1 January 2024 to 31 December 2024**										
**Age group**	**East Midlands**	**East of England**	**London**	**North East**	**North West**	**South East**	**South West**	**West Midlands**	**Yorkshire and Humber**	**Total**
less than 1 year	13	25	151	15	11	15	10	70	22	332
1 to 4 years	57	66	327	16	23	16	10	157	38	710
5 to 10 years	68	63	362	20	13	14	20	116	54	730
11 to 14 years	25	19	78	7	5	8	10	49	9	210
15 to 24 years	22	28	122	4	12	16	24	84	16	328
25 to 34 years	16	29	144	8	17	26	19	47	16	322
35 years and older	13	22	121	9	18	26	19	39	12	279
Total	214	252	1305	79	99	121	112	562	167	2911
**Laboratory confirmed cases of measles by age group and region in England: 1 January 2025 to 28 July 2025**										
**Age group**	**East Midlands**	**East of England**	**London**	**North East**	**North West**	**South East**	**South West**	**West Midlands**	**Yorkshire and Humber**	**Total**
less than 1 year	6	6	35	1	21	2	10	7	4	92
1 to 4 years	4	17	109	2	48	5	13	14	14	226
5 to 10 years	0	20	90	0	19	3	6	4	7	149
11 to 14 years	1	4	21	0	2	0	4	2	3	37
15 to 24 years	4	9	25	0	6	6	5	5	4	64
25 to 34 years	2	7	26	0	8	2	11	2	2	60
35 years and older	1	2	16	3	7	5	9	0	3	46
Total	18	65	322	6	111	23	58	34	37	674

Supplementary Table 2: Demographic characteristics of the study sample

**Variable**	**Number (%)**
**Age groups, years**	
1-4	358 (9.5%)
5-9	514 (13.7%)
10-14	587 (15.6%)
15-19	773 (20.6%)
20-24	769 (20.5%)
25-30	757 (20.1%)
**Sex**	
Males	1645 (43.8%)
Females	2113 (56.2%)
**Region**	
East Midlands	90 (2.4%)
North East	487 (13.0%)
North West	1943 (51.7%)
South West	566 (15.1%)
Yorkshire and Humberside	672 (17.9%)

Supplementary Table 3: Geometric mean titers of OD of measles serum IgG of seropositive individuals by demographic factors

	Number	Geometric Mean OD (95% CI)	Adjusted geometric mean ratio (95% CI)	P value*
**Age, years**				
1-4	324	1.23 (1.15-1.32)	Reference	
5-9	462	0.80 (0.75-0.85)	0.65 (0.59-0.71)	<0.001
10-14	459	0.68 (0.64-0.72)	0.54 (0.50-0.60)	<0.001
15-19	603	0.61 (0.58-0.65)	0.49 (0.45-0.53)	<0.001
20-24	575	0.58 (0.55-0.61)	0.46 (0.42-0.50)	<0.001
25-30	604	0.73 (0.69-0.78)	0.51 (0.46-0.57)	<0.001
**Sex**				
Males	1294	0.72 (0.69-0.75)	0.99 (0.94-1.04)	0.576
Females	1733	0.71 (0.69-0.74)	Reference	
**Region**				
East Midlands	68	0.66 (0.55-0.80)	0.87 (0.73-1.32)	0.103
North East	394	0.67 (0.62-0.71)	0.80 (0.74-0.88)	<0.001
North West	1553	0.71 (0.68-0.73)	0.81 (0.74-0.88)	<0.001
South West	448	0.73 (0.69-0.78)	0.82 (0.74-0.91)	<0.001
Yorkshire and Humberside	564	0.77 (0.73-0.81)	Reference	

CI: confidence interval; IgG: immunoglobulin G; OD: Optical Density

* P values were obtained from a multiple linear regression model with Log10 OD of measles serum IgG as the dependent variable and age, sex, and region as independent variables. Exponentiating the log10 OD was employed to obtain the geometric mean titers.

Seropositive: Optical density >0.2 using the manufacturer's recommended cut-off values.

Supplementary Table 4: The mean (standard deviation) of the log10 OD of measles IgG serum antibody by age in the negative, low positive, and high positive distributions – mixture modelling

Age, years	Negative	Low Positive	High Positive
1-4	-2.638 (1.093)	-0.153 (0.270)	0.237 (0.145)
5-9	-2.638 (1.093)	-0.459 (0.349)	0.019 (0.216)
10-14	-2.638 (1.093)	-0.524 (0.385)	-0.044 (0.228)
15-19	-2.638 (1.093)	-0.690 (0.405)	-0.224 (0.314)
20-24	-2.638 (1.093)	-0.826 (0.441)	-0.286 (0.322)
25-30	-2.638 (1.093)	-0.557 (0.444)	-0.020 (0.288)

IgG: immunoglobulin G; OD: optical density

Data presented are mean and standard deviation in parenthesis

Supplementary Table 5: Percentage of individuals with negative measles serum IgG antibody by age group: comparison between manufacturer's OD cut-off value for seronegativity (OD<0.1) and values generated by mixture modelling

Age group, years	% Negative with manufacturer's cut-off OD <0.1	Mixture model seronegative estimates (95% CI)			
		% Negative from modelled distribution	Age-specific OD cut-off	% Negative with age-specific OD cut-off	% Negative with OD cut-off <0.03 for all ages
1-4	8.7% (6.1-11.9)	9.3% (6.5-13.1)	0.11	8.7% (6.1-11.9)	7.8% (5.4-11.0)
5-9	4.5% (2.9-6.5)	1.4% (0.6-3.2)	0.05	1.4% (0.6-2.7)	1.4% (0.6-2.7)
10-14	11.2% (8.9-14.0)	6.4% (4.2-9.5)	0.04	6.1% (4.4-8.3)	5.5% (3.8-7.5)
15-19	9.7% (7.8-11.9)	3.8% (2.4-6.0)	0.03	3.9% (2.7-5.4)	3.9% (2.7-5.4)
20-24	11.3% (9.2-13.7)	1.9% (1.0-3.7)	0.02	2.1% (1.2-3.3)	3.1% (2.1-4.5)
25-30	8.2% (6.4-10.3)	0.8% (0.3-2.4)	0.02	0.9% (0.4-1.8)	2.1% (1.3-3.3)

IgG: immunoglobulin G; OD: optical density

Supplementary Table 6: Geometric mean titers of OD of rubella serum IgG of seropositive individuals by demographic factors

	Number	Geometric Mean OD (95% CI)	Adjusted Geometric mean ratio (95% CI)	P value *
**Age, years**				
1-4	329	1.43 (1.35-1.52)	Reference	
5-9	487	0.86 (0.81-0.91)	0.60 (0.55-0.66)	<0.001
10-14	515	0.81 (0.77-0.86)	0.56 (0.51-0.61)	<0.001
15-19	673	0.71 (0.68-0.75)	0.49 (0.45-0.53)	<0.001
20-24	662	0.67 (0.64-0.71)	0.46 (0.42-0.50)	<0.001
25-30	695	0.96 (0.91-1.01)	0.95 (0.51-0.63)	<0.001
**Sex**				
Males	1438	0.84 (0.81-0.87)	0.99 (0.94-1.03)	0.574
Females	1928	0.85 (0.82-0.87)	Reference	
**Region**				
East Midlands	80	0.84 (0.73-0.98)	0.91 (0.78-1.06)	0.232
North East	445	0.75 (0.70-0.79)	0.76 (0.69-0.83)	<0.001
North West	1713	0.82 (0.80-0.85)	0.80 (0.74-0.86)	<0.001
South West	494	0.816 (0.77-0.87)	0.78 (0.71-0.86)	<0.001
Yorkshire and Humberside	629	0.99 (0.94-1.05)	Reference	

CI: confidence interval; IgG: immunoglobulin G; OD: Optical Density

* P values were obtained from a multiple linear regression model with Log10 OD of rubella serum IgG as the dependent variable; age, sex, and region as independent variables. Exponentiating the log10 OD was employed to obtain the geometric mean titers.

Seropositive: Optical density >0.2 using the manufacturer's recommended cut-off values.

Supplementary Table 6: Percentage of individuals with negative rubella serum IgG antibody by age group: comparison between manufacturer's OD cut-off value for seronegativity (OD<0.1) and values generated by mixture modelling

Age group, years	% Negative with manufacturer's cut-off OD <0.1	Mixture model seronegative estimates (95% CI)			
		% Negative from modelled distribution	Age-specific OD cut-off	% Negative with age-specific OD cut-off	% Negative with OD cut-off <0.02 for all ages
1-4	7.0% (4.7-10.0)	7.1% (4.8-10.3)	0.1	7.0% (4.7-10.0)	7.0% (4.7-10.0)
5-9	3.1% (1.9-4.9)	1.5% (0.7-3.2)	0.02	1.6% (0.7-2.9)	1.6% (0.7-2.9)
10-14	5.3% (3.7-7.3)	4.4% (3.0-6.4)	0.03	4.3% (2.8-6.1)	4.3% (2.8-6.1)
15-19	6.1% (4.6-7.9)	3.7% (2.6-5.3)	0.03	3.6% (2.5-5.1)	3.6% (2.5-5.1)
20-24	6.0% (4.5-7.8)	1.8% (1.0-3.1)	0.01	1.7% (0.9-2.8)	2.0% (1.1-3.1)
25-30	4.1% (2.9-5.7)	1.1% (0.5-2.3)	0.02	1.1% (0.5-2.0)	1.1% (0.5-2.0)

Supplementary table 8: Sensitivity analysis of effective reproduction number (Re) estimates

Percentage estimated to be susceptible to measles by age, and effective reproduction number (Re) estimates, in regions outside London in 2018, based on coverage data under different assumptions about the percentage of individuals without a documented MMR vaccination history who were vaccinated

A: Vaccine efficacy for MMR dose 1 = 0.95 and for MMR dose 2 = 0.9975

Age group in years	Percentage vaccinated among those with no record of vaccination		
	10%	25%	50%
	Percentage estimated to be susceptible to measles		
0-4	19.3	18.4	16.9
5-10	5.2	4.3	3.0
11-17	6.9	5.8	3.9
18-24	9.3	7.8	5.3
25-90	3.3	3.1	2.7
**Re**	**0.65**	**0.57**	**0.51**

B: A: Vaccine efficacy for MMR dose 1=0.95 and for MMR dose 2=0.96

Age group in years	Percentage vaccinated among those with no record of vaccination		
	10%	25%	50%
	Percentage estimated to be susceptible to measles		
0-4	19.3	18.4	16.9
5-10	8.6	7.8	6.5
11-17	10.2	9.1	7.4
18-24	12.2	10.9	8.6
25-90	3.5	3.3	3.0
**Re**	**1.12**	**1.00**	**0.80**

Supplementary figures

Supplementary figure 1: The distribution (%) of laboratory-confirmed measles cases by age and region, England, 2023-July 2025

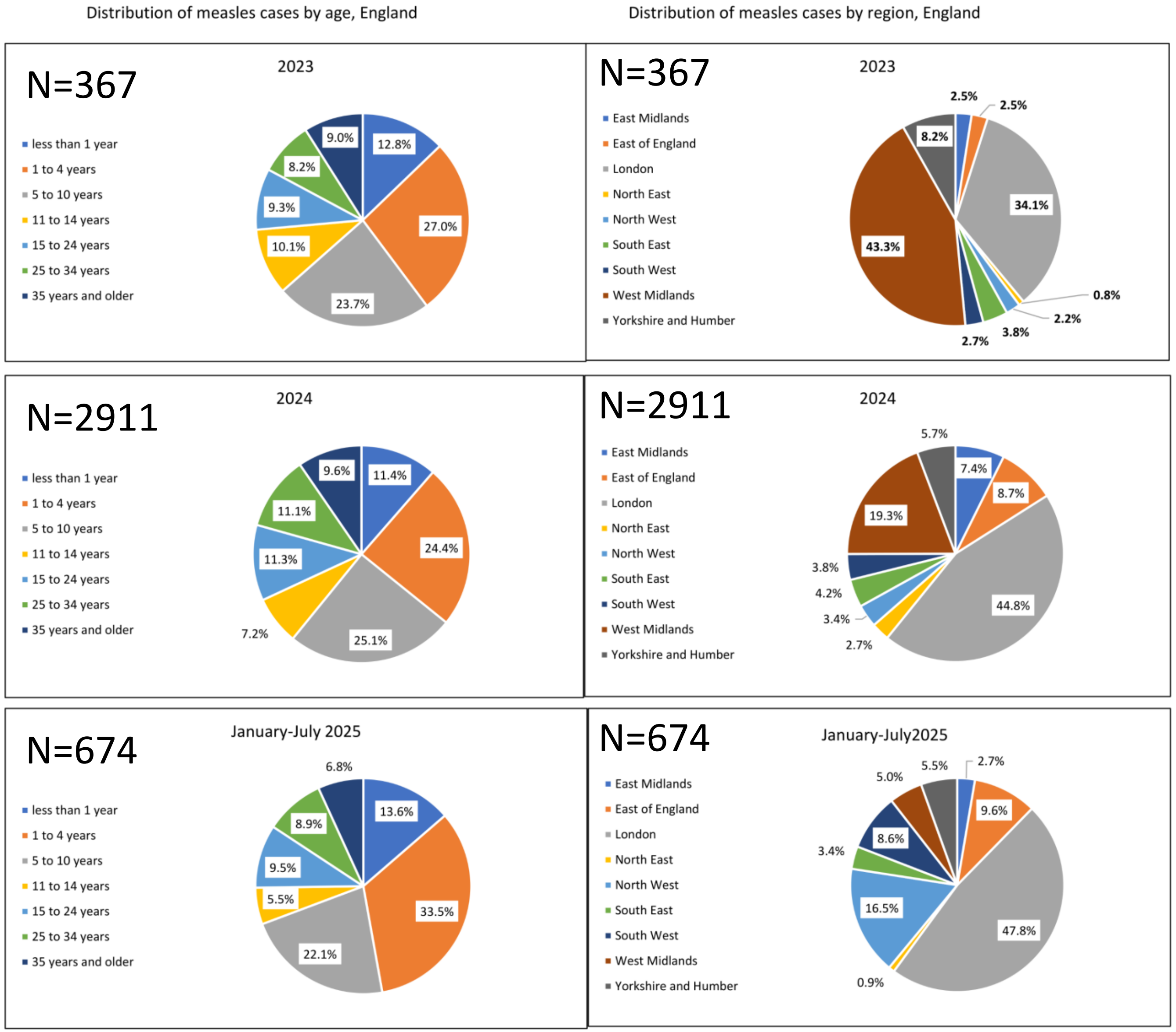

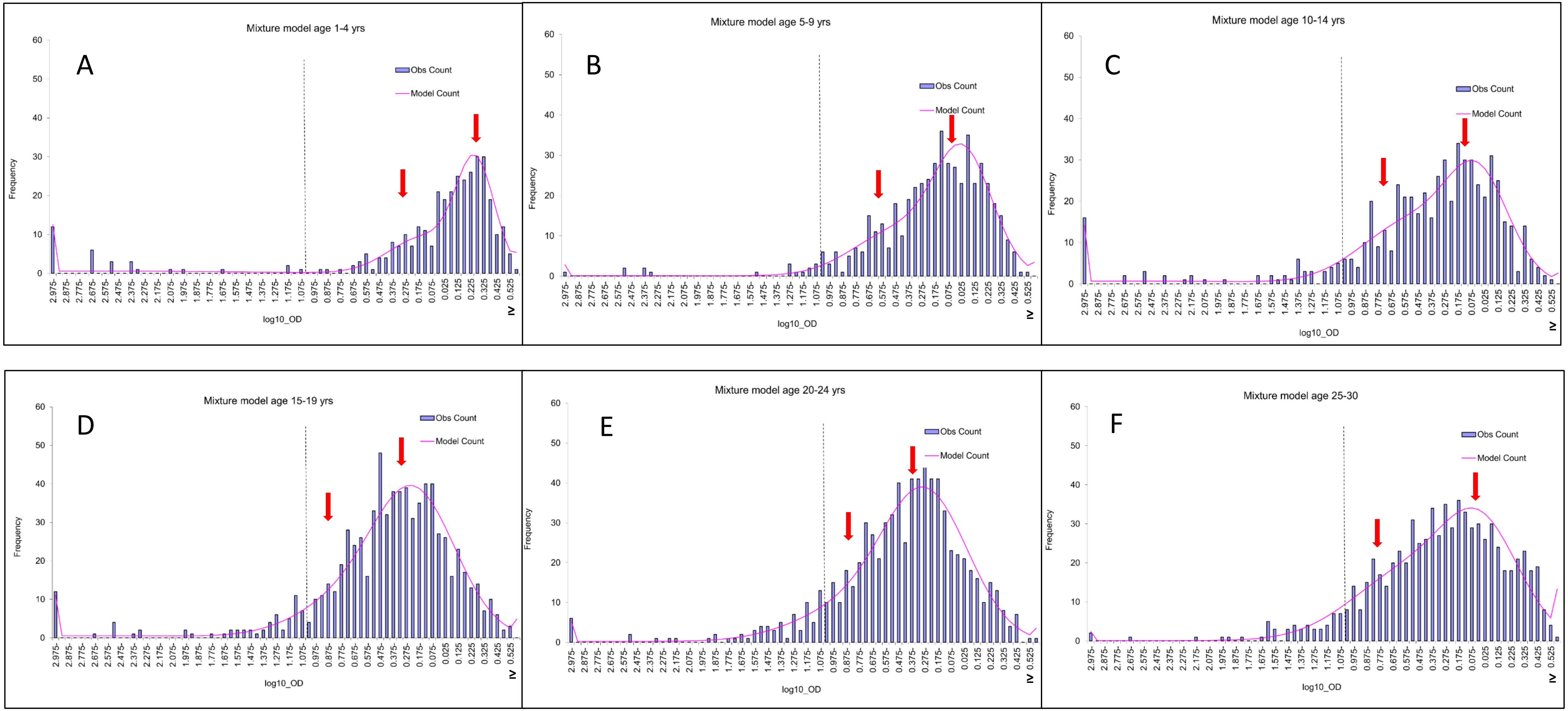

Supplementary Figure 2: Mixture model plots of log10 OD of measles IgG serum antibody level by age group

Panel A: age 1-4 years, B: age 5-9 years, C: age 10-14 years, D: age 15-19 years, E: age 20-24 years, F: age 25-30 years

The X-axis represents the log10 optical density (OD) of measles IgG measles serum antibody. The Y-axis represents the frequency. The bars represent the observed counts, and the pink lines represent the counts from mixture modelling. The dashed line is the cutoff to determine seronegativity based on the manufacturer's recommendation (OD<0.1, <-1.0 in log10 scale). The red arrows visually outline the potential of 2 positive distributions.

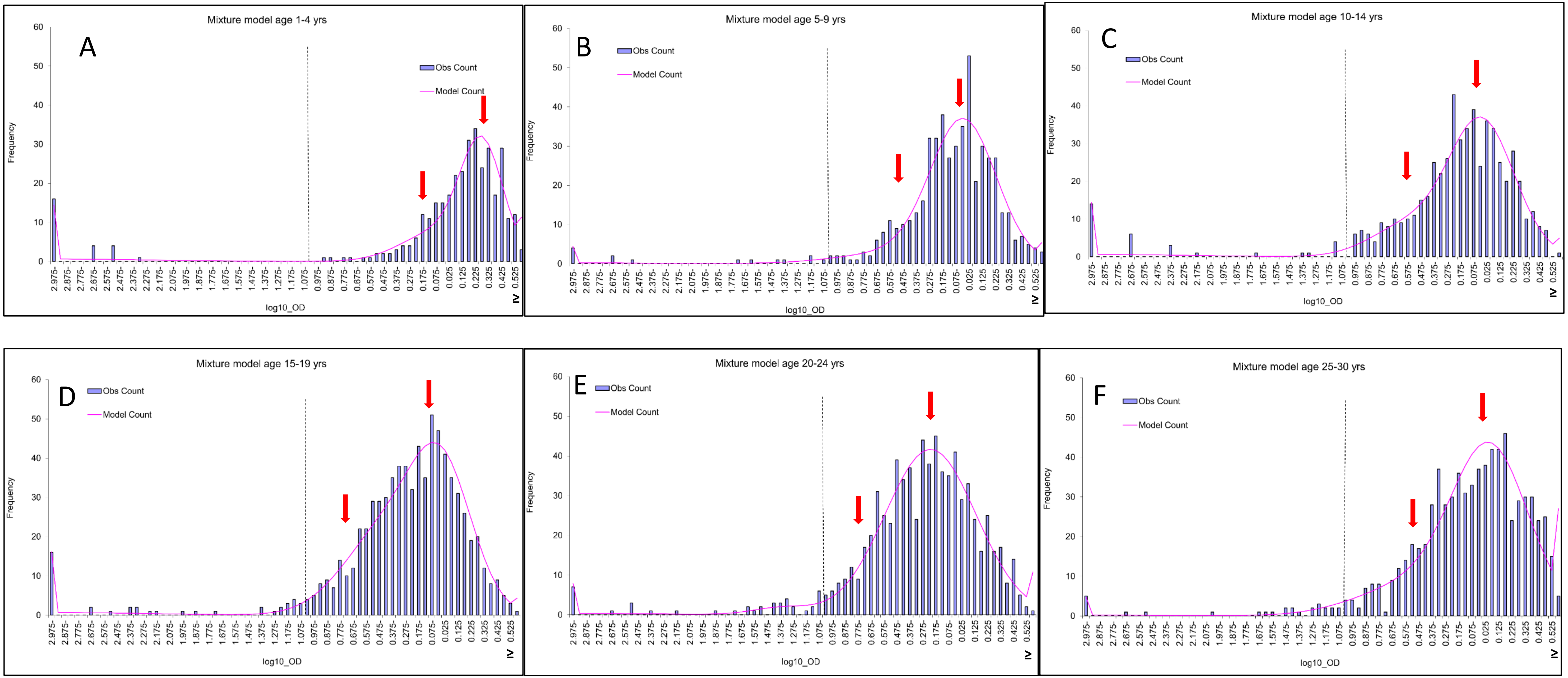

Supplementary Figure 3: Mixture model plots of log10 OD of rubella IgG serum antibody level by age group

Panel A: age 1-4 years, B: age 5-9 years, C: age 10-14 years, D: age 15-19 years, E: age 20-24 years, F: age 25-30 years

The X-axis represents the log10 optical density (OD) of rubella IgG serum antibody. The Y-axis represents the frequency. The bars represent the observed counts, and the pink lines represent the counts from mixture modelling. The dashed line is the cutoff to determine seronegativity based on the manufacturer’s recommendation (OD<0.1, <-1.0 in log10 scale). The red arrows outline visually the potential of 2 positive distributions.

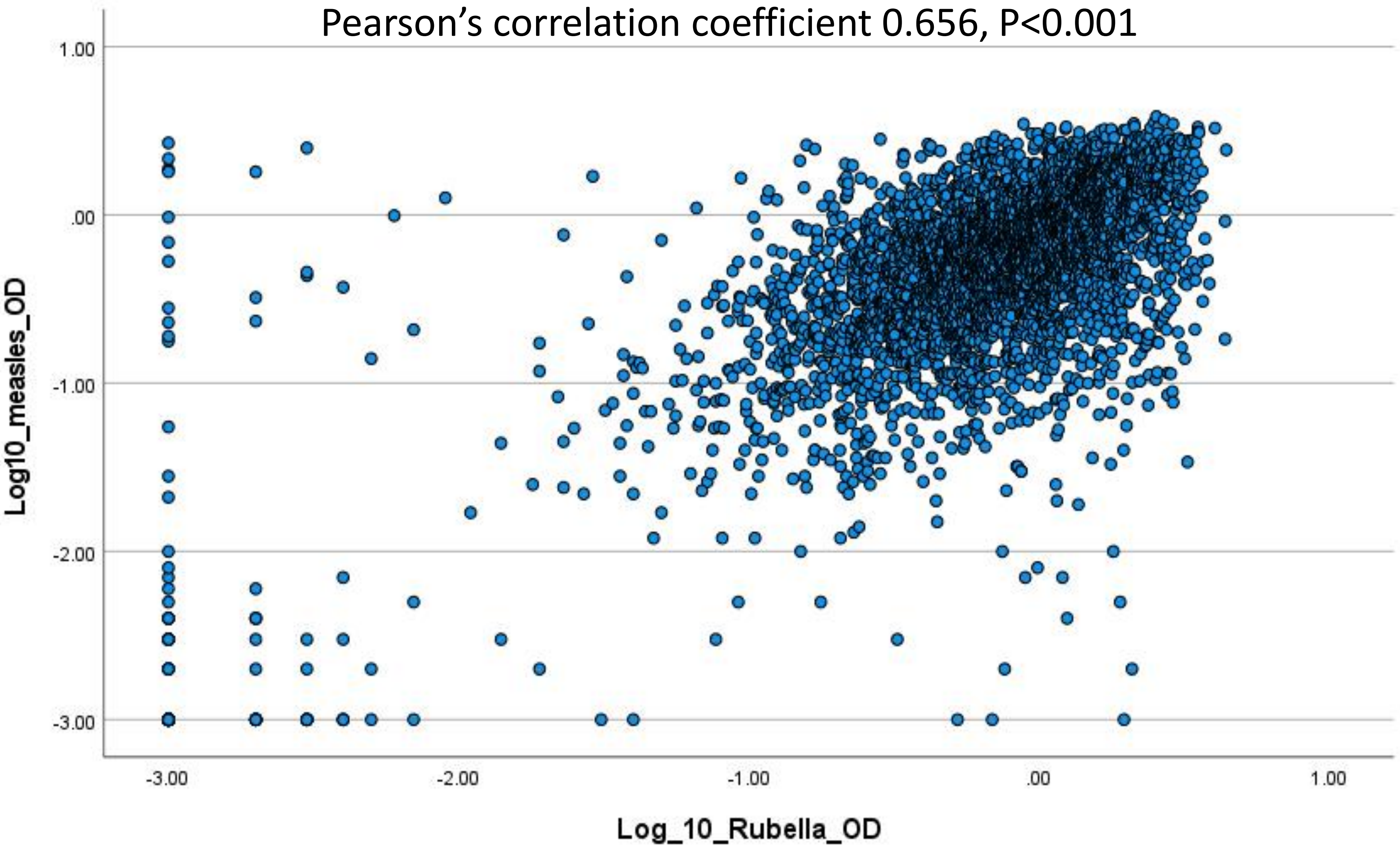

Supplementary Figure 4: Scatter plot of log10 OD of rubella and measles IgG antibodies

STATA code for mixture models

*STATA code to fit multi-normal model, allowing for 3 distributions with censoring

*y is the measles corrected delta OD. Those in our data with delta OD <0.001 have been replaced with 0.001 and values of >1000 replace by 2000

*we need a log scale for y

*create an indicator for censored values

generate cens=0

replace cens = 1 if y>1000

replace cens= -1 if y<=0.001

replace y = log10(y)

*program mix2normX written to fit multi-normal model

*want to model an overall negative, and low and high positive distributions

*input arguments are STARTING points generated using estimates from standard mixture models using stata fmm function

*(using stata function "fmm 3" to obtain overall the negative distribution starting estimates, and using function "fmm 2" by age group for low and high positive starting estimates)

*arguments are-

*mean and natural log SDs

*-for an OVERALL NEGATIVE model (mean1, logsigma1)

*- for each age group in LOW AND HIGH POSITIVE DISTRIBUTION (mean2_ageX, mean3_ageX, logsigma2_ageX, logsigma3_ageX)

*logit of the proportion negative and proportion in low positive groups by agegroup (loddsplow_ageX, loddsplow_ageX)

*proportion negative is as observed, proportion low is estimate from fitted mixture model, proportion high derived from negative and low

```
* program fits 3-normal distributions with censored data and transformed parameters to help with convergence

program mix2normX

args lnf mean1 logsigma1 mean2_age4 logsigma2_age4 mean3_age4 logsigma3_age4 mean2_age10 logsigma2_age10 mean3_age10 logsigma3_age10 mean2_age17 logsigma2_age17 mean3_age17 logsigma3_age17 mean2_age24 logsigma2_age24 mean3_age24 logsigma3_age24 mean2_age25 logsigma2_age25 mean3_age25 logsigma3_age25 loddspneg_age4 loddsplow_age4 loddspneg_age10 loddsplow_age10 loddspneg_age17 loddsplow_age17 loddspneg_age24 loddsplow_age24 loddspneg_age25 loddsplow_age25

tempvar s1 s2_age4 s3_age4 pn_age4 ppl_age4 pph_age4 s2_age10 s3_age10 pn_age10 ppl_age10 pph_age10 s2_age17 s3_age17 pn_age17 ppl_age17 pph_age17 s2_age24 s3_age24 pn_age24 ppl_age24 pph_age24 s2_age25 s3_age25 pn_age25 ppl_age25 pph_age25

qui g double `s1'=exp(`logsigma1')

qui g double `s2_age4'=exp(`logsigma2_age4')

qui g double `s3_age4'=exp(`logsigma3_age4')

qui g double `pn_age4'=exp(`loddspneg_age4')/(1+exp(`loddspneg_age4'))

qui g double `ppl_age4'=exp(`loddsplow_age4')/(1+exp(`loddsplow_age4'))

qui g double `pph_age4'=1-`ppl_age4'-`pn_age4'

qui g double `s2_age10'=exp(`logsigma2_age10')

qui g double `s3_age10'=exp(`logsigma3_age10')

qui g double `pn_age10'=exp(`loddspneg_age10')/(1+exp(`loddspneg_age10'))

qui g double `ppl_age10'=exp(`loddsplow_age10')/(1+exp(`loddsplow_age10'))

qui g double `pph_age10'=1-`ppl_age10'-`pn_age10'

qui g double `s2_age17'=exp(`logsigma2_age17')

qui g double `s3_age17'=exp(`logsigma3_age17')

qui g double `pn_age17'=exp(`loddspneg_age17')/(1+exp(`loddspneg_age17'))

qui g double `ppl_age17'=exp(`loddsplow_age17')/(1+exp(`loddsplow_age17'))
```

```
qui g double `pph_age17'=1-`ppl_age17'-`pn_age17'


qui g double `s2_age24'=exp(`logsigma2_age24')
qui g double `s3_age24'=exp(`logsigma3_age24')
qui g double `pn_age24'=exp(`loddspneg_age24')/(1+exp(`loddspneg_age24'))
qui g double `ppl_age24'=exp(`loddsplow_age24')/(1+exp(`loddsplow_age24'))
qui g double `pph_age24'=1-`ppl_age24'-`pn_age24'

qui g double `s2_age25'=exp(`logsigma2_age25')
qui g double `s3_age25'=exp(`logsigma3_age25')
qui g double `pn_age25'=exp(`loddspneg_age25')/(1+exp(`loddspneg_age25'))
qui g double `ppl_age25'=exp(`loddsplow_age25')/(1+exp(`loddsplow_age25'))
qui g double `pph_age25'=1-`ppl_age25'-`pn_age25'


qui replace `lnf'=ln( `pn_age4'*normalden(y,`mean1',`s1') + (`ppl_age4')*normalden(y,`mean2_age4',`s2_age4') + (`pph_age4')*normalden(y,`mean3_age4',`s3_age4')) if cens==0 & Agegroup=="1_4"

qui replace `lnf'=ln( `pn_age4'*normal( (y-`mean1')/`s1' ) + (`ppl_age4')*normal( (y-`mean2_age4')/`s2_age4') + (`pph_age4')*normal((y-`mean3_age4')/`s3_age4')) if cens==-1 & Agegroup=="1_4"

qui replace `lnf'=ln( `pn_age4'*(1-normal( (y-`mean1')/`s1') ) + (`ppl_age4')*(1-normal( (y-`mean2_age4')/`s2_age4')) + (`pph_age4')*(1-normal( (y-`mean3_age4')/`s3_age4'))) if cens==1 & Agegroup=="1_4"


qui replace `lnf'=ln( `pn_age10'*normalden(y,`mean1',`s1') + (`ppl_age10')*normalden(y,`mean2_age10',`s2_age10') + (`pph_age10')*normalden(y,`mean3_age10',`s3_age10')) if cens==0 & Agegroup=="5_10"

qui replace `lnf'=ln( `pn_age10'*normal( (y-`mean1')/`s1' ) + (`ppl_age10')*normal( (y-`mean2_age10')/`s2_age10') + (`pph_age10')*normal((y-`mean3_age10')/`s3_age10')) if cens==-1 & Agegroup=="5_10"

qui replace `lnf'=ln( `pn_age10'*(1-normal( (y-`mean1')/`s1') ) + (`ppl_age10')*(1-normal( (y-`mean2_age10')/`s2_age10')) + (`pph_age10')*(1-normal( (y-`mean3_age10')/`s3_age10'))) if cens==1 & Agegroup=="5_10"
```

```
qui replace `lnf'=ln( `pn_age17'*normalden(y,`mean1',`s1') +
(`ppl_age17')*normalden(y,`mean2_age17',`s2_age17') +
(`pph_age17')*normalden(y,`mean3_age17',`s3_age17')) if cens==0 & Agegroup=="11_17"

qui replace `lnf'=ln( `pn_age17'*normal( (y-`mean1')/`s1' ) + (`ppl_age17')*normal( (y-
`mean2_age17')/`s2_age17') + (`pph_age17')*normal((y-`mean3_age17')/`s3_age17')) if cens==-1 &
Agegroup=="11_17"

qui replace `lnf'=ln( `pn_age17'*(1-normal( (y-`mean1')/`s1') ) + (`ppl_age17')*(1-normal( (y-
`mean2_age17')/`s2_age17')) + (`pph_age17')*(1-normal( (y-`mean3_age17')/`s3_age17'))) if
cens==1 & Agegroup=="11_17"



qui replace `lnf'=ln( `pn_age24'*normalden(y,`mean1',`s1') +
(`ppl_age24')*normalden(y,`mean2_age24',`s2_age24') +
(`pph_age24')*normalden(y,`mean3_age24',`s3_age24')) if cens==0 & Agegroup=="18_24"

qui replace `lnf'=ln( `pn_age24'*normal( (y-`mean1')/`s1' ) + (`ppl_age24')*normal( (y-
`mean2_age24')/`s2_age24') + (`pph_age24')*normal((y-`mean3_age24')/`s3_age24')) if cens==-1 &
Agegroup=="18_24"

qui replace `lnf'=ln( `pn_age24'*(1-normal( (y-`mean1')/`s1') ) + (`ppl_age24')*(1-normal( (y-
`mean2_age24')/`s2_age24')) + (`pph_age24')*(1-normal( (y-`mean3_age24')/`s3_age24'))) if
cens==1 & Agegroup=="18_24"



qui replace `lnf'=ln( `pn_age25'*normalden(y,`mean1',`s1') +
(`ppl_age25')*normalden(y,`mean2_age25',`s2_age25') +
(`pph_age25')*normalden(y,`mean3_age25',`s3_age25')) if cens==0 & Agegroup=="25plus"

qui replace `lnf'=ln( `pn_age25'*normal( (y-`mean1')/`s1' ) + (`ppl_age25')*normal( (y-
`mean2_age25')/`s2_age25') + (`pph_age25')*normal((y-`mean3_age25')/`s3_age25')) if cens==-1 &
Agegroup=="25plus"

qui replace `lnf'=ln( `pn_age25'*(1-normal( (y-`mean1')/`s1') ) + (`ppl_age25')*(1-normal( (y-
`mean2_age25')/`s2_age25')) + (`pph_age25')*(1-normal( (y-`mean3_age25')/`s3_age25'))) if
cens==1 & Agegroup=="25plus"



end
```

*call function with initial starting estimates

matrix initial = [-2.687\ -1.0370469\ -0.242 \ -0.90765297 \ 0.2008 \ -1.793757464 \ -0.4741312 \ -0.985858921 \ -0.0022447 \ -1.508715993 \-0.7084277 \ -0.813447971 \ -0.1916323 \ -1.189271619 \-0.8062329 \ -0.810219614 \ -0.2751499 \ -1.117791836 \-0.5792634 \ -0.790447578 \ -0.0391786 \ -1.212901514 \ -2.466888144 \ -0.934942057 \ -4.007333185 \ -0.419060322 \ -2.887032857 \ -1.030269059 \ -3.36729583 \ -1.114781021 \ -3.838107322 \ -0.047563703]

ml model lf mix2normX /mean1 /logsigma1 /mean2_age4 /logsigma2_age4 /mean3_age4 /logsigma3_age4 /mean2_age10 /logsigma2_age10 /mean3_age10 /logsigma3_age10 /mean2_age17 /logsigma2_age17 /mean3_age17 /logsigma3_age17 /mean2_age24 /logsigma2_age24 /mean3_age24 /logsigma3_age24 /mean2_age25 /logsigma2_age25 /mean3_age25 /logsigma3_age25 /loddspneg_age4 /loddsplow_age4 /loddspneg_age10 /loddsplow_age10 /loddspneg_age17 /loddsplow_age17 /loddspneg_age24 /loddsplow_age24 /loddspneg_age25 /loddsplow_age25

ml check

ml init initial, copy

ml query

ml maximize

return list

matrix list r(table)

*DISPLAY OUTPUT OF FITTED MODEL

display "Overall NEGATIVE"

display "m1: " _b[mean1] " sd1: " exp(_b[logsigma1])

display "AGE 1-4"

```
display  "m2: "  _b[mean2_age4] " sd2: " exp(_b[logsigma2_age4])  " m3: "  _b[mean3_age4]  " sd3: " exp(_b[logsigma3_age4]) " p_neg: " (exp(_b[loddspneg_age4])/(1+exp(_b[loddspneg_age4]))) " p_low: " (exp(_b[loddsplow_age4])/(1+exp(_b[loddsplow_age4])))

display "AGE 5-10"

display "m2: "  _b[mean2_age10] " sd2: " exp(_b[logsigma2_age10]) " m3: "  _b[mean3_age10] " sd3: " exp(_b[logsigma3_age10]) " p_neg: " (exp(_b[loddspneg_age10])/(1+exp(_b[loddspneg_age10]))) " p_low: " (exp(_b[loddsplow_age10])/(1+exp(_b[loddsplow_age10])))

display "AGE 11-17"

display "m2: "  _b[mean2_age17] " sd2: " exp(_b[logsigma2_age17]) " m3: "  _b[mean3_age17] " sd3: " exp(_b[logsigma3_age17]) " p_neg: " (exp(_b[loddspneg_age17])/(1+exp(_b[loddspneg_age17]))) " p_low: " (exp(_b[loddsplow_age17])/(1+exp(_b[loddsplow_age17])))

display "AGE 18-24"

display "m2: "  _b[mean2_age24] " sd2: " exp(_b[logsigma2_age24]) " m3: "  _b[mean3_age24] " sd3: " exp(_b[logsigma3_age24]) " p_neg: " (exp(_b[loddspneg_age24])/(1+exp(_b[loddspneg_age24]))) " p_low: " (exp(_b[loddsplow_age24])/(1+exp(_b[loddsplow_age24])))

display "AGE 25 plus"

display "m2: "  _b[mean2_age25] " sd2: " exp(_b[logsigma2_age25]) " m3: "  _b[mean3_age25] " sd3: " exp(_b[logsigma3_age25]) " p_neg: " (exp(_b[loddspneg_age25])/(1+exp(_b[loddspneg_age25]))) " p_low: " (exp(_b[loddsplow_age25])/(1+exp(_b[loddsplow_age25])))
```
